# Extracellular Vesicles as Biomarkers of Acute Graft-vs.-Host Disease After Haploidentical Stem Cell Transplantation and Post-Transplant Cyclophosphamide

**DOI:** 10.3389/fimmu.2021.816231

**Published:** 2022-01-25

**Authors:** Giuseppe Lia, Clara Di Vito, Stefania Bruno, Marta Tapparo, Lucia Brunello, Armando Santoro, Jacopo Mariotti, Stefania Bramanti, Elisa Zaghi, Michela Calvi, Lorenzo Comba, Martina Fascì, Luisa Giaccone, Giovanni Camussi, Eileen M. Boyle, Luca Castagna, Andrea Evangelista, Domenico Mavilio, Benedetto Bruno

**Affiliations:** ^1^Division of Hematology, Department of Oncology, A.O.U. Città della Salute e della Scienza di Torino, Torino, Italy; ^2^Department of Molecular Biotechnology and Health Sciences, University of Torino, Torino, Italy; ^3^Unit of Clinical and Experimental Immunology, IRCCS Humanitas Research Hospital, Rozzano, Italy; ^4^Department of Medical Biotechnologies and Translational Medicine (BioMeTra), University of Milan, Milan, Italy; ^5^Department of Medical Sciences, Molecular Biotechnology Center, University of Torino, Torino, Italy; ^6^Bone Marrow Transplant Unit, IRCCS Humanitas Research Hospital, Rozzano, Italy; ^7^Division of Hematology and Medical Oncology, New York University Grossman School of Medicine, Perlmutter Cancer Center, New York University (NYU) Langone Health, New York, NY, United States; ^8^Clinical Epidemiology, A.O.U. Città della Salute e della Scienza di Torino, Torino, Italy

**Keywords:** extracellular vesicles, biomarkers, acute GvHD, haploidentical, correlation, miRNA

## Abstract

Even with high-dose post-transplant cyclophosphamide (PT-Cy) which was initially introduced for graft-versus-host disease (GvHD) prevention in the setting of HLA-haploidentical transplantation, both acute and chronic GvHDs remain a major clinical challenge. Despite improvements in the understanding of the pathogenesis of both acute and chronic GvHDs, reliable biomarkers that predict their onset have yet to be identified. We recently studied the potential correlation between extracellular vesicles (EVs) and the onset of acute (a)GvHD in transplant recipients from related and unrelated donors. In the present study, we further investigated the role of the expression profile of membrane proteins and their microRNA (miRNA) cargo (miRNA100, miRNA155, and miRNA194) in predicting the onset of aGvHD in haploidentical transplant recipients with PT-Cy. Thirty-two consecutive patients were included. We evaluated the expression profile of EVs, by flow cytometry, and their miRNA cargo, by real-time PCR, at baseline, prior, and at different time points following transplant. Using logistic regression and Cox proportional hazard models, a significant association between expression profiles of antigens such as CD146, CD31, CD140a, CD120a, CD26, CD144, and CD30 on EVs, and their miRNA cargo with the onset of aGvHD was observed. Moreover, we also investigated a potential correlation between EV expression profile and cargo with plasma biomarkers (e.g., ST2, sTNFR1, and REG3a) that had been associated with aGVHD previously. This analysis showed that the combination of CD146, sTNFR1, and miR100 or miR194 strongly correlated with the onset of aGvHD (AUROC >0.975). A large prospective multicenter study is currently in progress to validate our findings.

## Introduction

Hematopoietic cell transplantation (HCT) represents a potentially curative strategy for several hematological malignancies. In recent years, the use of post-transplant cyclophosphamide (PT-Cy) as graft-vs.-host disease (GvHD) prophylaxis led to a considerable expansion of haploidentical transplants (Haplo-HCT) with remarkable clinical outcomes (1). However, both acute and chronic GvHDs remain life-threatening complications (2, 3). To predict their onset and develop preemptive interventions, the identification of reliable biomarkers still represents an unmet need. It is widely assumed that the combination of a profound cytokine imbalance and donor alloreactive T-cells plays a major role in the pathogenesis of acute GvHD (aGvHD) (3, 4). Several systemic biomarkers, including micro(mi)RNAs (i.e., miR155, miR100, miR194, miR423, miR199a) (5–9), suppression of tumorigenicity 2 (ST2), tumor necrosis factor receptor 1 (TNFR1), and organ-specific biomarkers, such as regenerating islet-derived protein 3 alpha (REG3a), hepatocyte growth factor (HGF), and elafin, have been investigated as potential biomarkers of aGvHD in various biological fluids (10–13). Unfortunately, to date, none of these biomarkers have been able to universally predict either risk or severity of developing GvHD.

Extracellular vesicles (EVs) have recently been reported as a promising group of circulating biological biomarkers (14–16). EVs are cell-derived membranous structures containing different biomolecules, including nucleic acids, proteins, lipids, and carbohydrates. They play a major role in intercellular communication by transferring proteins, bioactive lipids, and miRNA to recipient cells (17–19). Increasing research on EVs has demonstrated that EVs are involved in many pathophysiological processes and that they might be exploited as biomarkers of several pathological conditions (20). Moreover, EVs can be isolated easily from body fluids, including blood and urine, in a minimally invasive manner. Our group recently reported that the expression of CD146, CD31, and CD140a on their surface significantly correlated with the risk of developing acute GvHD in HLA-identical HCT (21). To further investigate the role of EVs as an aGvHD biomarker, we hereby report the same analysis in the setting of Haplo-HCT with PT-Cy. Moreover, given the role of miR100, miR155, and miR194 in endothelial damage, inflammation, and neovascularization which are also key factors in the pathogenesis of aGVHD, we evaluated their expression level in EVs.

## Materials and Methods

### Patients, Transplant Characteristics, and Graft-vs.-Host Disease

Thirty-two consecutive patients who underwent a Haplo-HCT from related donors were enrolled at the Bone Marrow Transplant Unit, Humanitas Cancer Center, Humanitas Research Hospital in Rozzano, Milan, Italy. Patients and donors signed an informed consent, and all study procedures were conducted in accordance with the Declaration of Helsinki. Patient, disease, and transplant characteristics are summarized in **Table 1**.

**Table 1:** Patient and transplant characteristics.

	Number (%)
Patients	32
Median age, years (range)	41 (21–66)
Male	17 (53%)
**DISEASE**	
Hodgkin lymphoma	17 (53%)
Non-Hodgkin lymphoma	11 (34%)
Acute lymphoblastic leukemia	2 (6%)
Chronic lymphocytic leukemia	1 (3%)
Acute myeloid leukemia	1 (3%)
**Myeloablative conditioning**	
TBF	3/32 (9%)
**Reduced intensity conditioning (RIC)/non-myeloablative conditioning**	
Baltimore	22/32 (69%)
ONC005	6/32 (19)%
TBF RIC	1/32 (3)%
**Stem cell source**	
Bone marrow	31/32 (97%)
Peripheral blood stem cells	1/32 (3%)
**GvHD prophylaxis**	
Pt-Cy + tacrolimus + MMF	22/32 (69%)
Pt-Cy + CyA+ MMF	10/32 (31%)
**aGvHD grades II–IV**	7 (21.88%)
Median day of onset (range)	41 (33–90)
**aGvHD grades III–IV**	1 (17%)

TBF, thiotepa (5 mg/kg; days -6, -5) - fludarabine (50 mg/m^2^; days -4, -3, -2) - busulfan (30 mg/kg; days -4, -3, -2); Baltimore = fludarabine (30 mg/m^2^; days -6, -5, -4, -3, -2) – cyclophosphamide (14.5 mg/kg; days -6, -5), total body irradiation (200 cGy), ONC005 = thiotepa (5 mg/kg twice a day; day -6) - fludarabine (30 mg/m^2^; days -5; -4, -3, -2) - cyclophosphamide (30 mg/kg; days -5); TBF RIC = thiotepa (5 mg/kg; days -6, -5) - fludarabine (50 mg/m^2^; days -4, -3, -2) - busulfan (3.2 mg/kg; days -4, -3); PT-Cy = post-transplant cyclophosphamide; MMF= mycophenolic acid; CyA = cyclosporin A.

GvHD prophylaxis consisted of PT-Cy 50 mg/kg on days +3 and +4, tacrolimus and/or cyclosporin A, and mycophenolate mofetil (MMF) from day +5 post-transplant. Additionally, granulocyte colony-stimulating factor (G-CSF) was started on day +5. Disease response was defined according to the European Bone Marrow Transplantation (EBMT) Group criteria. Acute GvHD was graded according to Glucksberg score.

### Plasma Sample Collection

Peripheral blood was drawn on lithium-heparin, from both donors and recipients before transplant (day -6) and from the recipients after a median of 0, 3, 7, 14, 21, 30, 45, and days and 2, 2.5, and 3 months following transplant. Plasma samples were obtained after mononuclear cell separation by density gradient centrifugation (Lympholyte, Cedarlane, Burlington, Canada) and stored at −80°C until use (22).

### Extracellular Vesicle Precipitation and Characterization

**Figure 1:**
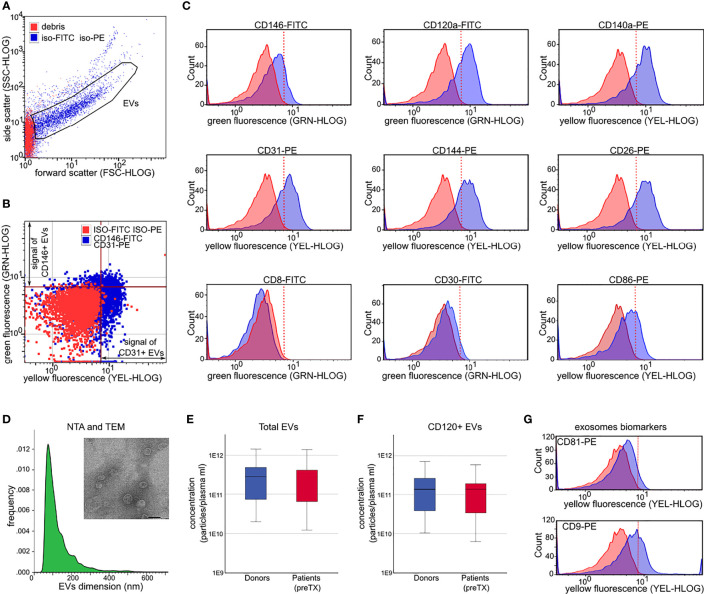
Extracellular vesicle (EV) characterization by light scattering and fluorescence. **(A)** Forward and side scatter dot plots of EVs analyzed after incubation with non-immune isotypic FITC and PE-IgG (iso-FITC/iso-PE, negative controls, blue dots). Red dots represent debris. **(B)** Representative fluorescence dot plots showing EV fluorescence after incubation with non-immune isotypic FITC and PE-IgG (negative controls, red dots), and after incubation with anti-CD146-FITC and anti-CD31-PE (blue dots). The red line marks the threshold to discriminate the positive FITC (green fluorescence) and the positive PE fluorescence (yellow fluorescence) signal from the background. **(C)** Representative histograms showing the shift in fluorescence after incubation of EVs with the indicated antibodies (blue peaks) with respect to isotypic control (FMO, red peaks). **(D)** Donor EV dimension histograms by nanoparticle tracking analysis. Inset: representative image of EVs detected by transmission electron microscopy (magnification ×60,000; scale bar 50 nm). **(E, F)** Plasma concentration of total EVs **(E)** and concentration of CD120+ EVs **(F)** in donors (blue) and in patients prior to transplant (preTX, red). **(G)** Representative flow cytometry histograms showing the shift in fluorescence after incubation of donor EVs with anti-CD9-PE and anti-CD81-PE (exosomes biomarkers). EVs = gated region; red line marks threshold to discriminate positive florescence signal from background.

For each sample, 1 ml of plasma was thawed on ice and centrifuged at 2,000 × g at 4°C for 40 min to remove platelet contamination. EVs were then precipitated as previously described (21). After precipitation, EVs were resuspended in 150 μl of Roswell Park Memorial Institute (RPMI) medium supplemented with penicillin, streptomycin, and amphotericin B, plus 10% of dimethyl sulfoxide (DMSO), and stored at -80°C until use. EV size and concentration were assessed by nanoparticle tracking (NTA) analysis (21). The presence of EVs on precipitated samples was confirmed by transmission electron microscopy. EVs were left to adhere to 200-mesh Nickel Formvar^®^ carbon-coated grids (Electron Microscopy Sciences) for 10 min. Grids were then washed with phosphate-buffered saline (PBS), fixed with 2.5% glutaraldehyde containing 2% sucrose, negatively stained with NanoVan^®^ (Nanoprobes), and observed by JEOL JEM-1400 Flash electron microscope (Tokyo, Japan). The presence and percentage of exosomes in our precipitated EV samples were measured by flow cytometry using CD9 and CD81 phycoerythrin (PE)-conjugated antibodies (**Figure 1**).

### Flow Cytometry Analysis

EVs were characterized by flow cytometry using fluorescein isothiocyanate (FITC) or phycoerythrin (PE)-conjugated antibodies, investigating the expression of 14 EV membrane proteins (**Supplementary Table 1**). Mouse non-immune isotypic FITC or PE IgGs (Miltenyi Biotec, Bergisch Gladbach, Germany) were used as negative controls. Incubation of tagged antibodies (0.8–1.5 µl) and EVs (3 × 10^9^ particles), flow cytometry acquisition on a Guava Instrument (Guava easyCyte™ 8, Merck Millipore, Billerica, MA, USA), and gate setting were performed as previously described (21).

For each marker, a total of 5,000–10,000 events were acquired at low speed (repeated 2–4 times) to determine a) the mean fluorescence intensity (MFI) and b) the percentage and concentration of positive EVs (**Figure 1** and **Supplementary Figure 2**). Concentrations of positive EVs for given markers were obtained by multiplying the frequency of positive events and total EV concentration. Data were analyzed using the guavaSoft InCyte 2.5 program.

### miRNA Extraction

miRNas were extracted from EVs by TRIzol LS (Thermo Fisher Scientific, Waltham, MA, USA) according to the manufacturer’s instructions. Briefly, 70 µl of EV suspension was diluted in 180 μl of PBS (pH 7.4) and lysed by adding 750 µl of TRIzol LS. Subsequently, 200 µl of chloroform was added and samples were centrifuged at 12,000 × g at 4°C for 15 min to allow phase separation. The upper aqueous phase was then transferred, and 750 µl of 100% ethanol was added to allow the RNAs precipitation. MiRNAs were then purified by a miRNeasy Mini Kit (Qiagen, Hilden, Germany), according to the manufacturer’s instruction. RNA concentrations were assessed using a NanoDrop 2000 spectrophotometer (Thermo Scientific, Waltham, MA, USA).

### miRNA Reverse Transcription and Real-Time PCR Quantification

MiRNAs were reverse transcribed to cDNA using a miScript II RT Kit (Qiagen). Briefly, 60 ng of input RNA from all samples was reverse transcribed at 37°C for 1 h in the presence of 2 units of Bacteroides Heparinase I (NEB, Ipswich, MA, USA) in a final volume of 15 µl (23).

The expression of miR100, miR155, miR194 was then assessed by semiquantitative real-time PCR (qRT-PCR) using the miScript SYBR Green PCR Kit (Qiagen). RNU6b and miR92b were used as housekeeping reference genes to normalize qRT-PCR outputs. All samples were run at least in triplicate using 3 ng of cDNA for each reaction in a final volume of 10 µl. qRT-PCR was performed as follows: 15 min at 95°C; 15 s at 94°C, 30 s at 55°C, 30 s at 70°C for 52 cycles, and finalized by a dissociation curve with a 5-s dwell time for each 0.5°C increment.

Data were expressed as relative to healthy donor quantification (RQ) using the ΔΔCt method. miR92b was used as stable miRNA reference given its lower intra-patient expression variability in comparison to RNU6b (**Supplementary Figure 3**) (24).

### Enzyme-Linked Immunosorbent Assay

Soluble forms of human TNFR1, ST2, and REG3a were assessed in plasma samples using commercially available sandwich enzyme-linked immunosorbent assays (ELISA; R&D Systems Europe, Abingdon Science Park, Abingdon, UK). Plasma samples were diluted in 1% bovine serum albumin in PBS (1:15 for TNFR1, 1:15-1:60 for ST2, and 1:200 for REG3a).

Plasma concentrations of TNFR1, ST2, and REG3a were determined according to the manufacturer’s protocol in both donors and recipients before transplant and at different time points post-transplant.

### Statistical Analyses

Cumulative incidences of aGvHD were calculated from the date of transplant to the date of onset. The estimations were performed considering relapse or death from any cause as competing event as described by Gooley et al. (25) Patients alive without GvHD were censored at the last available follow-up time point. The effects of repeated measurements of each marker on incidence of aGvHD were analyzed dividing the follow-up of each patient in a period of 7 days without considering pre-transplant measurements and the first week after. Patients were classified by presence/absence of aGvHD during each period. In case of more than one measurement for a given marker in the same 7-day period, the analysis was performed considering the mean value. Thus, the probability of developing aGvHD in each period with respect to marker levels, evaluated as absolute measure and as proportional change from pre-transplant value [expressed as (biomarker value - pre-transplant value)/pre-transplant value], was calculated by the logistic regression model (LRM). The effects on aGvHD incidence were reported as standardized odds ratio (OR), indicating the effect for a 1-standard deviation (SD) increase for a given variable per 1-point increase (relative increase of 100%) and corresponding p value for statistical significance. Analyses were based on repeated measurements on the same patient; ORs were estimated checking the standard errors with the Huber–White Sandwich Estimator. Moreover, for sensitivity analysis, Cox proportional hazard models (CM) for aGvHD were estimated using EV parameters at each time point as a time-varying covariate and reporting the hazard ratios (HRs) for a 1-SD increase. The discrimination ability on predicting the aGvHD of single biomarkers was evaluated by performing univariable logistic regression models including as independent variable the repeated measurements within the 100-day period of each marker and estimating the univariable area under the receiver operating characteristics (AUROCs). Multivariable models were then estimated to include most predictive markers performing a backward selection by Akaike information criterion selecting a maximum of three biomarkers due to the small number of aGVHD events. Correlation between markers was measured using the Spearman correlation coefficient, and markers most correlated (r >0.30) were included in the models. Finally, for each model, multivariable AUROCs were estimated. Due to repeated measures in the same patient, standard errors of ORs were adjusted using the Huber–White sandwich estimator. All statistical analyses were performed using STATA 15 (StataCorp LP) and SPSS Statistics 25 (IBM SPSS Statistics).

## Results

### Acute GvHD

Acute GvHD requiring systemic therapy was observed in 7/32 (22%) of patients with a median day of onset at +41 (range +33–+90) (**Table 1**). The cumulative incidence of grade II–IV aGvHD at day 100 was 21.9% (95% confidence interval (CI): 9.6–37.2%) (**Supplementary Figure 1**).

### EV Characterization and Correlation With aGvHD

*Fluorescence*—CD146 fluorescence change was significantly associated with increased risk of aGvHD by both logistic regression and Cox regression models (OR 2.93 p < 0.001, and HR 2.69 p = 0.009, respectively). CD30 fluorescence change was associated with an increased risk of aGVHD only by logistic regression (OR 1.58 p = 0.042). Even though CD25 fluorescence was associated with increased risk of aGvHD, its significance should be considered minimal given the overall very low fluorescence levels of this marker (**Table 2**).

**Table 2:** Association between EV surface biomarker level and aGvHD.

Marker	Type	Logistic regression				Cox model			
		Change		Absolute		Change		Absolute	
		OR	p	OR	p	HR	p	HR	p
Total EV conc.		**.53**	**.01**	**.70**	**.045**	.83	.465	1.43	.407
CD120a	Fluo.	1.50	.193	1.33	.026	1.14	.632	.83	.645
	Conc.	**.58**	**.018**	.76	.129	.89	.632	1.50	.309
CD140a	Fluo.	1.12	.627	1.05	.685	.90	.688	.75	.5
	Conc.	**.55**	**.013**	.73	.066	.80	.374	1.29	.555
CD44	Fluo.	.80	.508	.89	.38	1.17	.544	1.53	.25
	Conc.	.71	.194	.73	.068	1.21	.469	1.87	.083
CD26	Fluo.	1.12	.642	1.06	.575	1.18	.501	1.18	.643
	Conc.	**.59**	**.017**	.74	.065	.91	.694	1.61	.264
CD146	Fluo.	**2.93**	**<.001**	**1.25**	**.048**	**2.69**	**.009**	1.26	.586
	Conc.	.58	.096	.76	.176	.80	.423	1.12	.76
CD31	Fluo.	.92	.656	.97	.825	.89	.636	.87	.735
	Conc.	**.62**	**.047**	.83	.288	.81	.453	1.37	.461
CD106	Fluo.	1.21	.48	1.07	.671	1.34	.296	1.45	.321
	Conc.	.72	.133	.74	.125	.99	.977	1.61	.228
KRT18	Fluo.	1.23	.454	1.04	.729	1.20	.474	1.01	.981
	Conc.	.92	.677	.88	.483	1.12	.662	1.45	.364
CD30	Fluo.	**1.58**	**.042**	1.12	.37	1.53	.185	1.02	.969
	Conc.	1.40	.051	.98	.894	**2.37**	**.018**	**2.79**	**.029**
CD144	Fluo.	.92	.691	1.05	.696	.81	.433	.90	.793
	Conc.	**.70**	**.034**	**.48**	**.004**	1.52	.322	.75	.291
CD25	Fluo.	**1.87**	**.046**	.94	.588	**1.93**	**.044**	1.18	.725
	Conc.	1.07	.793	1.05	.785	1.43	.198	.88	.796
CD86	Fluo.	1.17	.464	.97	.876	1.20	.455	.93	.895
	Conc.	.76	.264	.88	.37	.79	.379	.88	.808
CD8	Fluo.	.88	.578	1.15	.277	1.15	.55	.98	.955
	Conc.	.79	.211	1.09	.545	1.25	.418	.58	.175
CD138	Fluo.	.90	.762	.93	.695	.99	.977	1.09	.852
	Conc.	.64	.066	.72	.054	.96	.881	1.51	.336

EV, extracellular vesicle; FLUO., fluorescence; HR, hazard ratio; OR, odd ratio; CONC., concentration of positive EVs (particles/plasma ml).

Marker analysis by 7-day time periods (logistic regression analysis), and by a time-varying approach (Cox model-proportional hazard model). Significant odd and hazard ratios (OR and HR respectively) are in bold.

**Figure 2:**
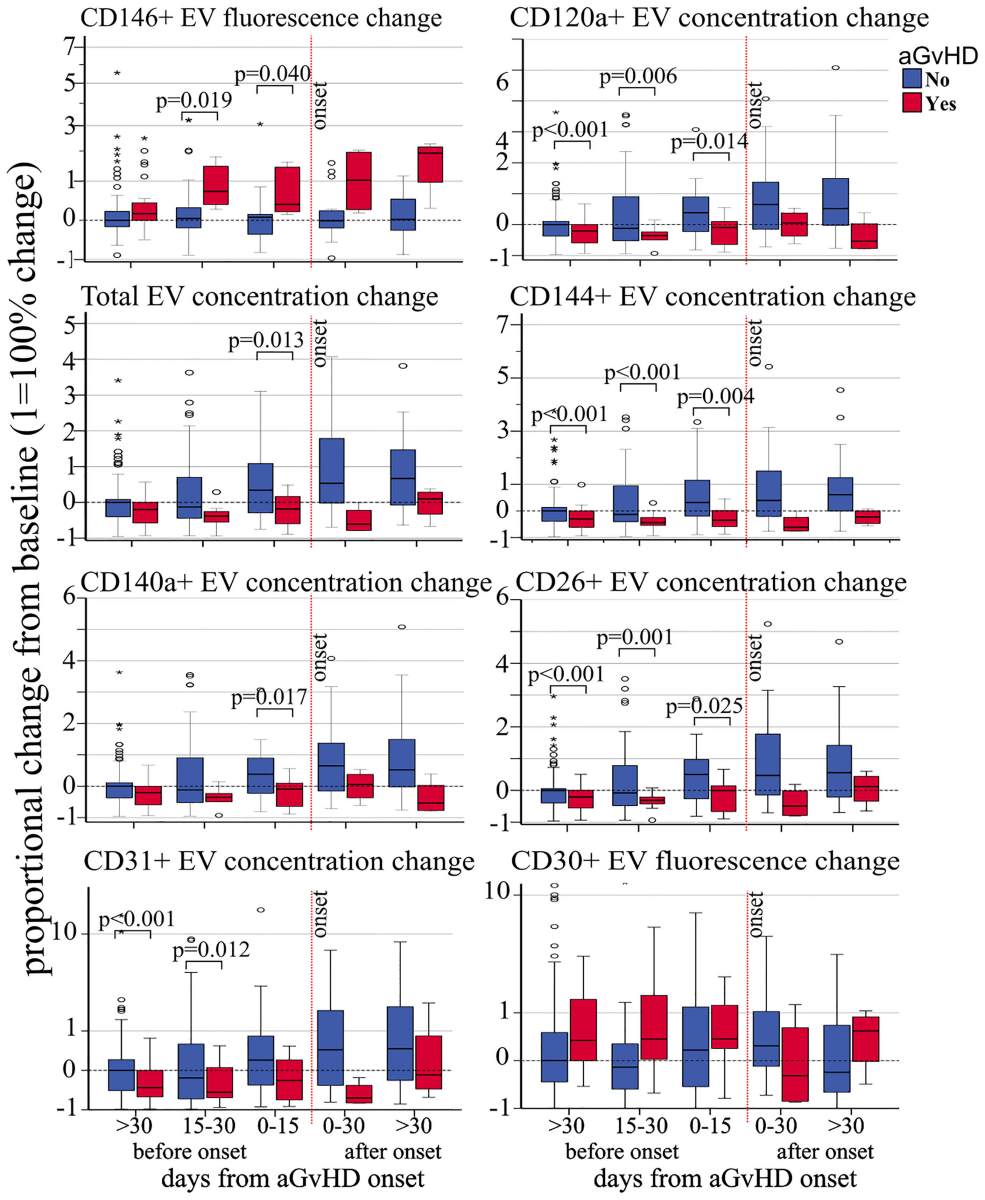
Impact of acute GvHD onset on the kinetics of EV membrane protein expression. Proportional change of fluorescence levels of CD146, total EV concentration, CD140a^+^ EV concentration and CD31^+^ EV concentration (left side, from top to bottom), CD120a^+^ EV concentration, CD144^+^ EV concentration, CD26^+^ EV concentration, and fluorescence levels of CD30 from precipitated EVs at different time points before and after aGvHD onset and compared to the pre-transplant baseline values. Dashed black line: pre-transplant levels; dashed red line: time of aGvHD onset; circle and star dots represent outliers (>1.5 box length from median) and extreme values (>3 box length from median), respectively. Significant mean differences before the onset (p ≤ 0.05) between patients with aGvHD (red) and without (blue) are indicated.

*Proportional concentration change—*proportional concentration changes in total EVs (OR 0.53, p = 0.01) and in CD120a (OR 0.58, p = 0.018), CD140a (OR 0.55, p = 0.013), CD26 (OR 0.59, p = 0.017), CD31 (OR 0.62, p = 0.047), and CD144 (OR 0.70, p = 0.034) were significantly associated with decreased risk of aGvHD (**Table 2**). Moreover, proportional concentration changes in CD30 were associated with increased risk of aGvHD (OR 1.40, p = 0.051). By contrast, we did not observe any correlation between CD44, CD106, KRT18, CD86, CD8, and CD138 and aGvHD (**Table 2****)**. Our findings also showed that these changes were detectable several weeks before the onset of aGvHD (**Figure 2**).

### miRNA Quantification and Correlation With aGVHD

**Figure 3:**
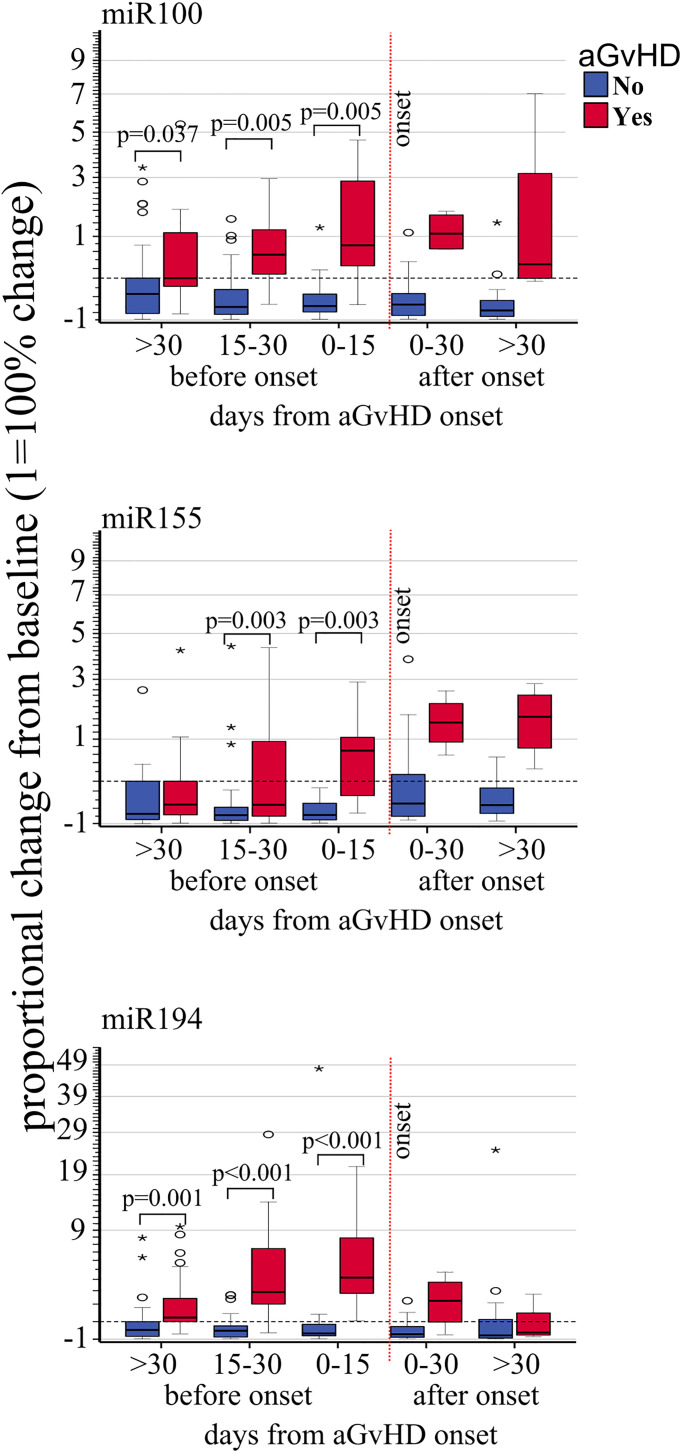
Impact of acute GvHD onset on the kinetics of EV miRNA. Proportional change of miR100, miR155, and miR194 (from top to bottom) quantified by real-rime PCR from precipitated EVs at different time points before and after aGvHD onset and compared to the pre-transplant baseline values. Dash black line: pre-transplant levels; dashed red line: time of aGvHD onset; circle and star dots represent outliers (>1.5 box length from median) and extreme values (>3 box length from median), respectively. Significant mean differences before the onset (p ≤ 0.05) between patients with aGvHD (red) and without (blue) are indicated.

Expression changes of miR100 (OR 3.90 p <.001, HR 2.63, p = 0.001), miR155 (OR 1.84, p = 0.008, HR 2.43, p = 0.002), and miR194 (OR 2.68 p < 0.001, HR 2.99, p = 0.001) were correlated with increased risk of developing aGVHD by both logistic regression and Cox regression models (**Table 3A**). Moreover, proportional expression change analyses showed that all three miRNAs significantly increased before aGVHD onset (**Figure 3**).

**Table 3:** Association between acute GvHD and EV-derived miRNAs and plasmatic biomarker levels.

A								
Marker	Logistic regression				Cox model			
miRNA EV	Change		Absolute		Change		Absolute	
	OR	p	OR	p	HR	p	HR	p
miR100	**3.90**	**<.001**	**1.84**	**<.001**	**2.63**	**.001**	**2.61**	**.014**
miR155	**1.84**	**.008**	**1.41**	**.012**	**2.43**	**.002**	**2.93**	**.01**
miR194	**2.68**	**<.001**	**1.39**	**.013**	**2.99**	**.001**	**2.24**	**.022**
**B**								
**Marker**	**Logistic regression**				**Cox model**			
**Plasma proteins**	**Change**		**Absolute**		**Change**		**Absolute**	
	**OR**	**p**	**OR**	**p**	**HR**	**p**	**HR**	**p**
ST2	1.04	.227	1.55	.058	1.03	.156	1.64	.053
sTNFR1	**1.47**	**.041**	1.56	.151	**1.42**	**.005**	1.41	.117
REG3a	.77	.086	1.17	.425	.89	.636	1.18	.446

EV, extracellular vesicle; HR, hazard ratio; OR, odd ratio.

Marker analysis by 7-day time periods (logistic regression analysis), and by a time-varying approach (Cox model-proportional hazard model). Significant odd and hazard ratios (OR and HR respectively) are in bold.

### Plasma Level Measurement of Soluble Biomarkers and Correlation With aGVHD

**Figure 4:**
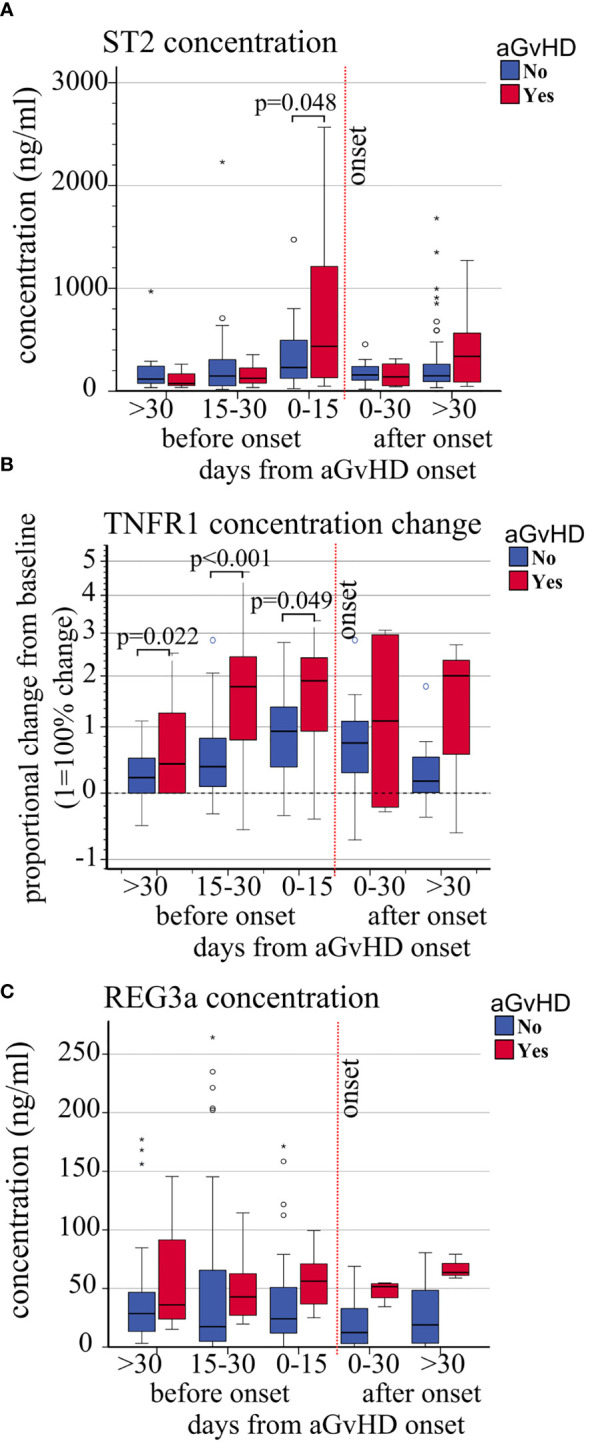
Impact of aGvHD onset on the circulating levels of ST2, sTNFR1, and REG3a. Variations of **(A)** absolute ST2 plasma level concentrations (ng/ml), **(B)** sTNFR1 relative plasma concentrations, and **(C)** absolute REG3a plasma level concentrations (ng/ml) from pre-transplant baseline levels, in patients with (red) and without (blue) aGvHD at different time points before and after aGvHD onset. Dashed black line: pre-transplant levels; dashed red line: time of aGvHD onset; circle and star-shaped dots represent outliers (>1.5 box length from median) and extreme values (>3 box length from median), respectively. Significant mean differences before the onset (p ≤ 0.05) between patients with aGvHD (red) and without (blue) are indicated.

The absolute concentration (ng/ml) and concentration change of sTNFR1 (OR 1.47, p = 0.041, and HR 1.42, p = 0.005, respectively) were significantly associated with increased risk of aGVHD whereas a trend was observed with ST2 (OR 1.55 p = 0.058, HR 1.64, p = 0.053) (**Table 3B**). No association was observed between REG3a and aGvHD. Moreover, the mean plasmatic concentrations and concentration changes of ST2 and sTNFR1 from day+15 were significantly different in patients with or without aGvHD (**Figures 4** and **Supplementary Figure 4**).

### Biomarker Performance

**Figure 5:**
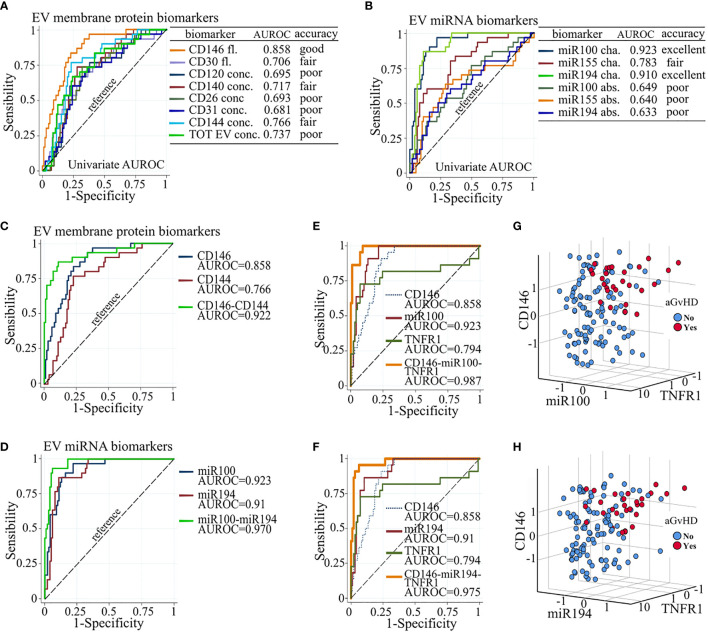
Biomarker combination improves aGvHD prediction. Individual AUROC curve analysis of aGVHD diagnostic performance of **(A)** EV membrane protein and **(B)** EV miRNA biomarkers. Individual and multivariate AUROC curve analysis of aGvHD diagnostic performance of **(C)** CD146 and CD144; **(D)** miR100 and miR194; **(E)** CD146, miR100, and sTNFR1; and **(F)** CD146, miR194, and sTNFR1. AUROC, area under the receiver operating characteristics; dot line, reference line; Fl., fluorescence; conc., concentration; cha., proportional change from basal; abs., absolute value. Three-D scatter plot of standardized proportional change from baseline of **(G)** CD146 fluorescence, miR100 expression, and sTNFR1 plasma concentration, and of **(H)** CD146 fluorescence, miR194 expression, and sTNFR1 plasma concentration, in patients with (red) and without (blue) aGvHD.

AUROC curves showed that miR100 and miR194 displayed excellent discriminating performance in separating patients with or without aGvHD (individual AUROC 0.923 and 0.91, respectively) and that CD146 had good performance (AUROC 0.858), whereas the other biomarkers that correlated with aGVHD had either poor or fair performance (**Figures 5A, B****)**. Based on the Akaike information criteria, the combination of CD146 and CD144 among the EV membrane proteins, and miR100 and miR194, had high multivariate AUROC, 0.922 and 0.970, respectively (**Figures 5C, D****)**. Two triplet combinations, CD146, miR100, sTNFR1 (combination 1) and CD146, miR194, sTNFR1 (combination 2), (**Figures 5E, F****)** showed the highest AUROC, 0.987 and 0.975, respectively, and allowed to better discriminate patients with or without aGvHD (**Figures 5G, H****)**.

## Discussion

Biomarkers that could reliably predict the onset of aGvHD and ensure preemptive interventions are lacking, meaning that diagnosis and treatment rely on clinical signs and symptoms and tissue biopsies only. Although several molecules/proteins have been investigated (16, 21, 26–28), in the present study, we evaluated the antigenic profile and miRNA cargo of EVs in the setting of Haplo-HCT using PT-Cy as GvHD prophylaxis.

Overall, we confirm that the risk of developing aGvHD was directly associated with CD146 expression and inversely correlated with total EV concentration and CD31 and CD140a concentrations. CD146 (or melanoma cell adhesion molecule) is a marker of activated endothelial cells, also expressed by CCR5+ T helper 17 (Th17) cells which expand during gastrointestinal aGVHD (29, 30). Moreover, this T-cell population plays an important role in many autoimmune diseases and inflammatory conditions (31, 32). CD31 (or platelet/EC adhesion molecule) is also a marker of endothelial activation. CD31 prevents lymphocyte hyperreactivity by increasing the activation threshold of the T-cell receptor (33). Its immune-regulatory role has been clearly demonstrated in murine models where CD31-deficient mice show a pronounced tumor rejection and excessive immune reactivity (34, 35). Decreased levels of CD31 in patients with aGvHD may indicate the loss of its protective role against inflammation and detrimental immunological attacks. We also observed a parallel reduction of the EV concentration of CD140a, also known as platelet-derived growth factor receptor-alpha (PDGFR-α), which is instrumental in the migration of fibroblasts and wound healing (36). Pro-inflammatory tumor necrosis factor-alpha (TNF-α) levels are usually higher in patients with aGvHD (37) and play a pivotal role in both initiating and amplifying aGvHD (38). TNF-α also decreases the expression of PDGFR-α after fibroblast injury, and its increased levels during aGvHD could consequently reduce fibroblast activation and tissue recovery (39). Interestingly, we observed that VE-cadherin, also known as CD144, is downregulated, which could be correlated with the increased TNF-α levels observed before aGvHD (40, 41). Increased TNF-α during aGvHD promotes vascular permeability by internalization and degradation of VE-cadherin, a calcium-dependent transmembrane cell–cell adhesion molecule, which regulates the formation of adherent junctions between endothelial cells, thus ensuring the physiological permeability and endothelial structure (42–45). We also investigated the EV surface expression of CD120a and its circulating soluble form TNFR1 (46). Increased levels of plasmatic sTNFR1 were shown to be associated with aGvHD (10, 47–49). This could result from the increased activity of the receptor sheddase (i.e., TACE) which proteolytically cleaves the TNFR1 ectodomain (50). Moreover, the increased TNFR1 sheddase activity may partly explain our observation that the concentration of CD120a^+^ EVs is reduced in aGvHD (51). All these findings are highly suggestive of endothelial activation.

Significant concentration changes of antigen expression indicating T-cell activation were also observed before the onset of aGvHD. CD30 is a type 1 transmembrane receptor of the TNF/nerve growth factor receptor family (TNFRSF8). It acts as a co-stimulatory molecule in T-cell responses and identifies/defines proliferating T-cell populations induced by allogeneic antigens (52). We observed a significant increase of CD30 in patients developing aGvHD as previously reported (52–54). By contrast, we observed a decreased level of circulating CD26. CD26, also known as dipeptidyl peptidase IV (DPPIV), is a cell surface glycoprotein enzyme associated with immune regulation, signal transduction, and apoptosis of several cell types (55). CD26 has been also described as a marker of T-cell activation and as an important regulator of inflammation (56–59). It accumulates in inflamed tissues and in target organs of aGvHD (60, 61). This may explain in part why its expression on EVs is reduced during aGVHD as seen in several autoimmune and other inflammatory conditions (62, 63).

The miRNA cargo of EVs is pivotal for their functions in both physiological and pathological conditions. In particular, we evaluated miR100, miR155, and miR194, given their association with inflammatory conditions and aGvHD, although their expression levels in EVs have not yet been explored. MiR100 has been described as an important player in regulating the inflammatory neovascularization during GvHD (8). In our patient cohort, the miR100 cargo gradually increased after transplant until the onset of aGvHD. However, its absolute levels remained lower compared to healthy donors and patients without aGvHD (**Supplementary Figure 5**). MiR155 is a critical regulator of inflammation and of innate and adaptive immune responses (64, 65). It has been reported that miR155 modulates aGvHD by driving a proinflammatory Th1 phenotype and by facilitating T-cell expansion, migration, and effector functions (64). Moreover, miR155 is upregulated in donor-derived T-cells in both preclinical mouse models and patients with GvHD. Its downmodulation, with synthetic anti-miR155, decreased aGvHD severity and prolonged survival in mice (5). MiR194 has been found significantly upregulated in patients who would later develop aGvHD. Of note, pathway prediction analyses suggest that these miRNAs regulate critical pathways in aGvHD pathogenesis, such as JAK-STAT, CXCL3, and TGFβ signaling. They could potentially become therapeutical targets (6). Our findings confirm their potential pivotal role in the development of aGvHD after haploidentical transplantation.

Plasma concentrations of sTNFR1, ST2, and REG3a have been extensively studied (66). We confirm previous findings showing the correlation of ST2 and sTNFR1 with aGvHD. We did not observe any correlation of REG3a. This, however, is likely due to the fact that only one of our patients developed gastrointestinal aGvHD. Finally, our ROC analysis showed that three of the studied biomarkers (miR100, miR194, and CD146) showed excellent or good performance (ROC > 0.8). By combining the most informative biomarkers, specificity, predictability, and diagnostic performance could increase. Combinations of CD146 fluorescence and CD144 concentration or miR100 and miR194 represent the minimal combinations that improve the diagnostic performance (multivariate ROC > 0.92). Combinations of plasma levels of sTNFR1, fluorescence of CD146, and miR100 or miR194 were the best combinations that significantly improved the diagnostic performance (multivariate ROC > 0.975) in discriminating patients with and without aGvHD.

Reproducibility and standardization are key to the development of clinically applicable biomarkers. Different methods of EV isolation and characterization may be employed. We used PEG precipitation to isolate EVs from our samples given the volume of starting material (<1 ml). Importantly, our analyses can be carried out in 24–48 h. Although ultracentrifugation techniques may be considered the gold standard for EV purification, they would be more difficult to standardize and be more expensive to run in a clinical laboratory (67–69).

Although our present observations are consistent with our previous findings (21), our cohort remains small in size, with relatively few cases of aGvHD, most of them affecting only the skin and being low in grade (one case III–IV aGvHD only). However, to further confirm our findings and to validate our model, a large multicenter prospective study including patients with different hematological malignancies and transplanted from different donor types has been designed and currently accruing. In summary, our report indicates a turbulence of significant dynamic changes in surface markers and miRNA cargo in plasma EVs that may specifically underlie events that precede the onset of aGvHD. They appear to mainly express endothelial injury and T-cell activation. Furthermore, our biomarker performance analyses suggest that combinations of EVs with other plasma biomarkers may reliably identify patients with incipient aGvHD.

## Data Availability Statement

The raw data supporting the conclusions of this article will be made available on request from the corresponding author.

## Author Contributions

BB, DM, and GC designed the study. GL, LB, CDV, and BB wrote the report. BB supervised the conduction of the study and data analyses. SBru, BB, and DM supervised the laboratory procedures. AS, JM, SBra, LCa, EMB and LG supervised the data collection, analyzed the data, and reviewed and assisted in writing the manuscript. GL, CDV, MT, EZ, MC, MF, and LCo undertook the experimental procedures. AE did the statistical analysis. All authors contributed to the article and approved the submitted version.

## Funding

This work was partly supported by Fondi di Ricerca Locale, Università degli Studi di Torino, Torino, Italy; by Fondazione EMN Italy Onlus (Fondazione European Myeloma Network Italy Onlus), Torino, Italy; by Fondazione Cariplo (2015/0603); by Associazione Italiana per la Ricerca sul Cancro (IG 2018-21567); by Italian Ministry of Health (Bando Ricerca Finalizzata); and by Intramural Research Funding of Istituto Clinico Humanitas. EZ is a recipient of two fellowships from the Associazione Italiana per la Ricerca sul Cancro (“Nella Orlandini” 2018/2019-20870 and “Giancarlo Iuri Amadio” 2020-24051). CDV was a recipient of the post-doctoral fellowships from the Fondazione Umberto Veronesi (2017-1464, 2018-1974, 2019-2563).

## Conflict of Interest

The authors declare that the research was conducted in the absence of any commercial or financial relationships that could be construed as a potential conflict of interest.

## Publisher’s Note

All claims expressed in this article are solely those of the authors and do not necessarily represent those of their affiliated organizations, or those of the publisher, the editors and the reviewers. Any product that may be evaluated in this article, or claim that may be made by its manufacturer, is not guaranteed or endorsed by the publisher.
