# Supplementary material for: Extracellular Vesicles as Biomarkers of Acute Graft-vs.-Host Disease After Haploidentical Stem Cell Transplantation and Post-Transplant Cyclophosphamide
Source: Front Immunol. 2022 Jan 25;12:816231. doi: 10.3389/fimmu.2021.816231 (PMC8821147; doi:10.3389/fimmu.2021.816231)

SUPPLEMENTARY MATERIALS

Extracellular Vesicles as Biomarkers of acute Graft-vs.-host Disease after Haploidentical Stem Cell Transplantation and Post-transplant Cyclophosphamide

Giuseppe Lia[1,2†], Clara Di Vito[3,4†], Stefania Bruno[5], Marta Tapparo[5], Lucia Brunello[1,2], Armando Santoro[6], Jacopo Mariotti[6], Stefania Bramanti[6], Elisa Zaghi[3], Michela Calvi[3,4], Lorenzo Comba[1,2], Martina Fascì[1,2], Luisa Giaccone[1,2], Giovanni Camussi[5], Eileen M. Boyle,[7] Luca Castagna[6], Andrea Evangelista[8], Domenico Mavilio[3,4], Benedetto Bruno[2,7]

Supplementary Tables

Supplementary Table 1. Panel of Markers used in Guava experiments.

Antigen	Company	Clone	Alternative Definition	Ligand/Receptor
**CD44**	Miltenyi Biotech, Bergisch Gladbach, Germany	REA690	H-CAM, Pgp-1, Hermes antigen, ECMRIII	Hyaluronin,osteopontin, fibronectin
**CD138**	BioLegend, San Diego, CA	MI15	Syndecan-1	Extra Cellular Matrix
**CD146**	Miltenyi Biotech, Bergisch Gladbach, Germany	541-10B2	MUC18, S-endo, MCAM, Mel-CAM	
**KRT18**	Abnova, Jhouzih St., Taipei, Taiwan	C-04	Keratin 18 Type I, Cytokeratin-18, CYK18	C-Cbl, TRADD, Collagen, type XVII, alpha 1, DNAJB6, Pinin
**CD120a**	Miltenyi Biotech, Bergisch Gladbach, Germany	REA252	TNFR-1, TNFRSF1A	TNFα, TNFβ
**CD8**	Miltenyi Biotech, Bergisch Gladbach, Germany	BW135/80	T8, Leu-2	MHC class I
**CD30**	Miltenyi Biotech, Bergisch Gladbach, Germany	KI-2	Ki-1, Ber-H2	CD153
**CD106**	Miltenyi Biotech, Bergisch Gladbach, Germany	REA269	VCAM-1, INCAM-110	CD49d/CD29, CD49d/β7
**CD25**	BioLegend, San Diego, CA	BC96	Tac, p55, IL-2Ra	IL-2
**CD31**	Miltenyi Biotech, Bergisch Gladbach, Germany	REA730	PECAM-1, endocam, GPIIa	CD31, CD138
**CD144**	R&D Systems, Minneapolis, MN	123413	VE-Cadherin, Cadherin-5	CD144, β-Catenin
**CD86**	Miltenyi Biotech, Bergisch Gladbach, Germany	REA968	B70, B7-2	CD28, CD152
**CD140a**	BioLegend, San Diego, CA	16A1	PDGFRA, PDGFRa	PDGF-A, PDGF-B, PDGF-C
**CD26**	Miltenyi Biotech, Bergisch Gladbach, Germany	FR10-11G9	Dipeptidyl peptidase-4	

Supplementary Figure Legends

Supplementary Figure 1. Cumulative incidence of acute II-IV GVHD at day +100 (21.9%; 95% confidence interval (CI): 9.6–37.2%).

Supplementary Figure 2. EV fluorescence histograms. Red areas: mean fluorescence intensity (MFI) of EVs incubated with the non-immune isotypic IgG tagged with FITC or PE; blue areas: MFI histograms of EVs incubated with a tagged antibody. Fluorescence signals (Fluo) of given markers were calculated by subtracting isotypic control MFI. Red dot lines mark the threshold to discriminate positive FITC (green fluorescence) and PE fluorescence (yellow fluorescence) signals from background.

Supplementary Figure 3.

MiR92b and RNU6b delta Ct from patient median Ct at different time-points after transplant (expression level stability of internal RNA molecules used for miRNAs expression level normalization).

Supplementary Figure 4.

Impact of aGvHD onset on the circulating levels of ST2, sTNFRI, and REG3a. Variations of absolute plasma level concentrations (ng/ml, left) and relative plasma concentrations from pre-transplant baseline levels (right) of (**A**) ST2, (**B**) sTNFRI, and (**C**) REG3a, in patients with (red) and without (blue) aGvHD at different time-intervals after transplant (pre-transplant, between days +7 and +14, and between days +15 and +28); Variations of absolute plasma level concentrations and relative plasma concentrations of (**D**) ST2, (**E**) sTNFRI, and (**F**) REG3a from pre-transplant baseline levels at different time points before and after aGvHD onset. Dashed black line: pre-transplant levels; Dashed line represents pre-transplant levels; dashed red line: time of aGvHD onset; circle and star-shaped dots represent outliers (>1.5 box length from median) and extreme values (>3 box length from median), respectively. Significative p-value ($p \leq 0.05$) for mean difference between patients with aGvHD (red) and without (blue) are indicated.

Supplementary Figure 5.

MiR100 level in EVs before the onset of aGvHD. Relative quantification (RQ) of miR100 in patients with (red) and without (blue) aGvHD versus healthy donors (gray) before transplant (preTX) and before the onset of aGvHD. Circle and star-shaped dots represent outliers (>1.5 box length from median) and extreme values (>3 box length from median), respectively.

Supplementary Figure 1

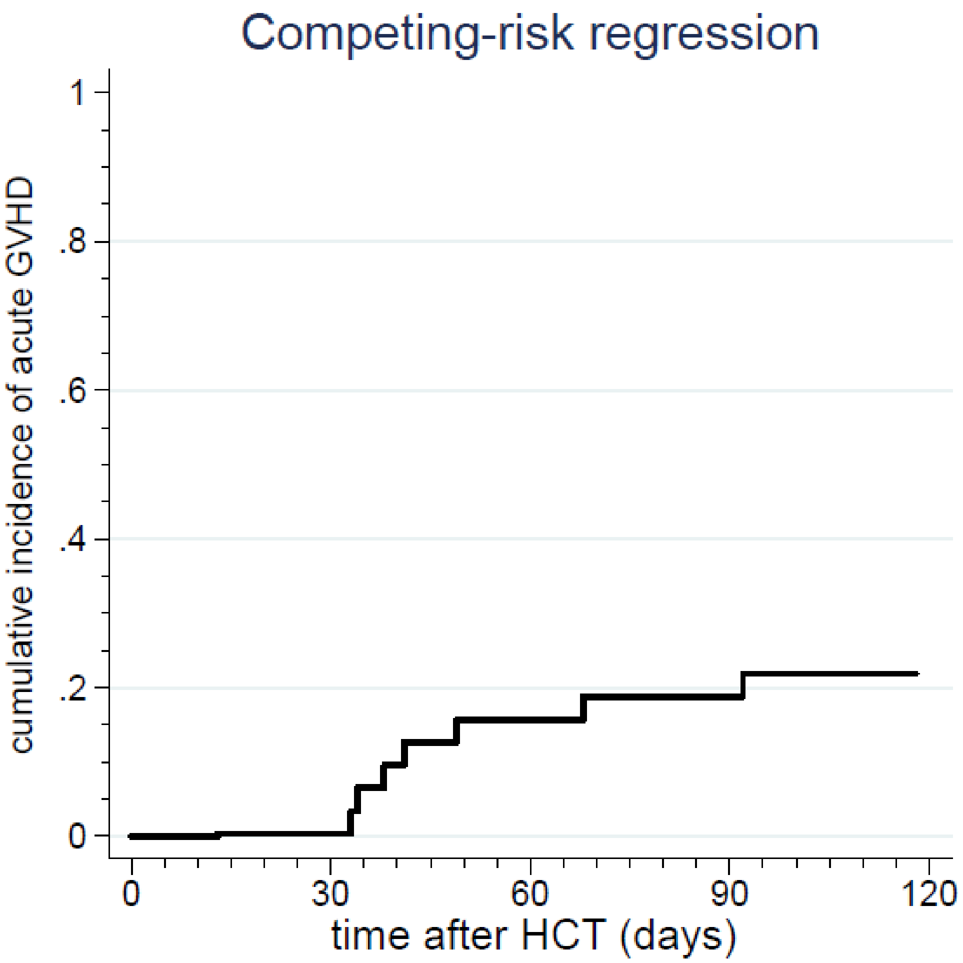

Supplementary Figure 2

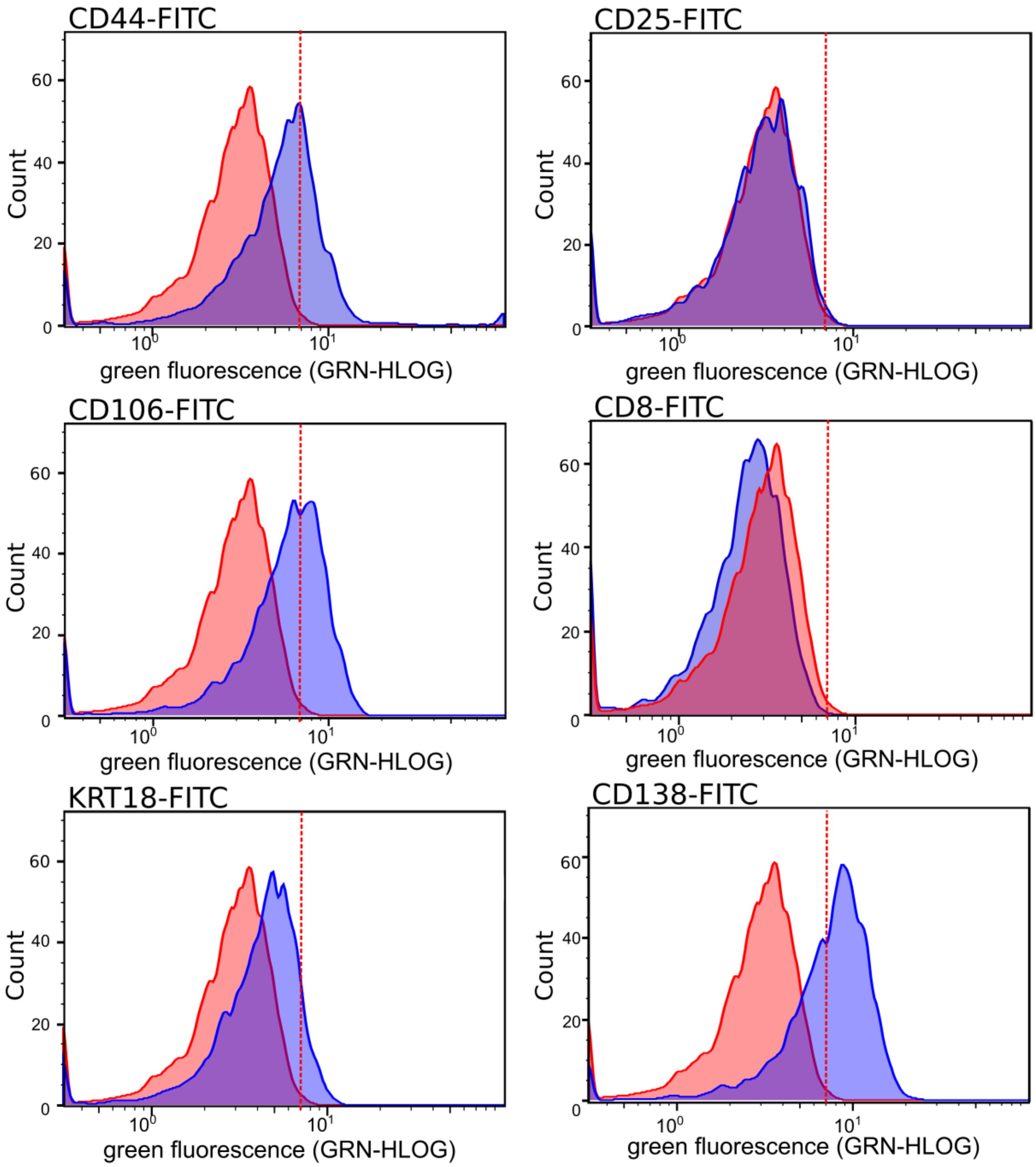

Supplementary Figure 3

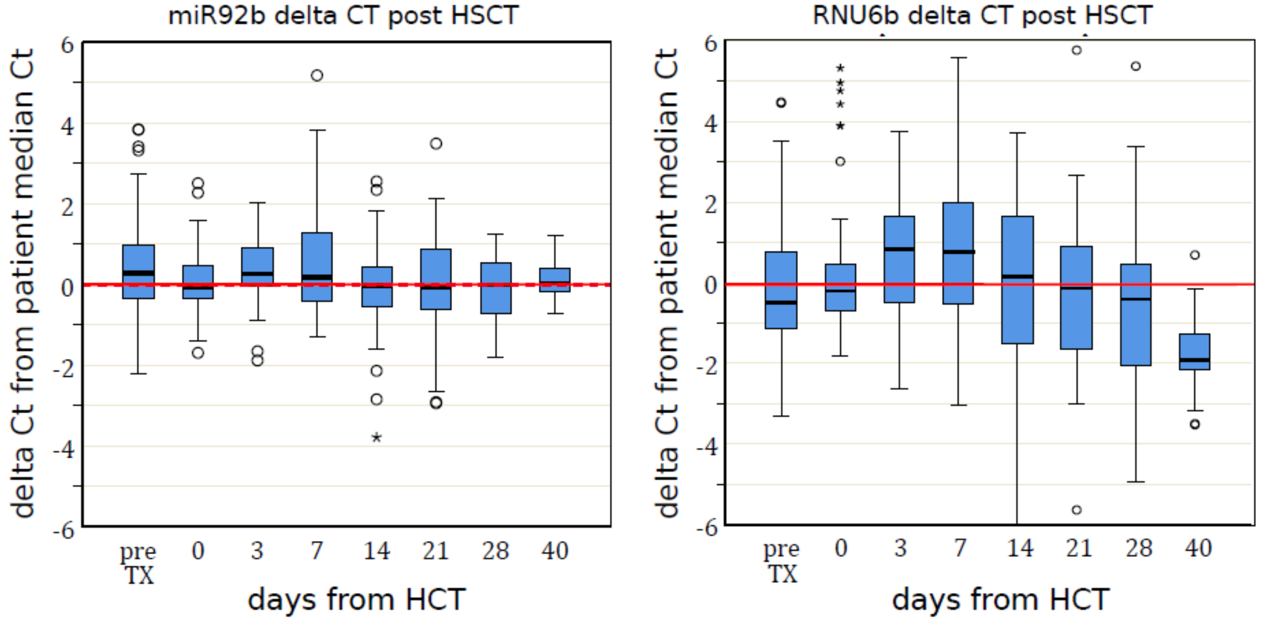

Supplementary Figure 4

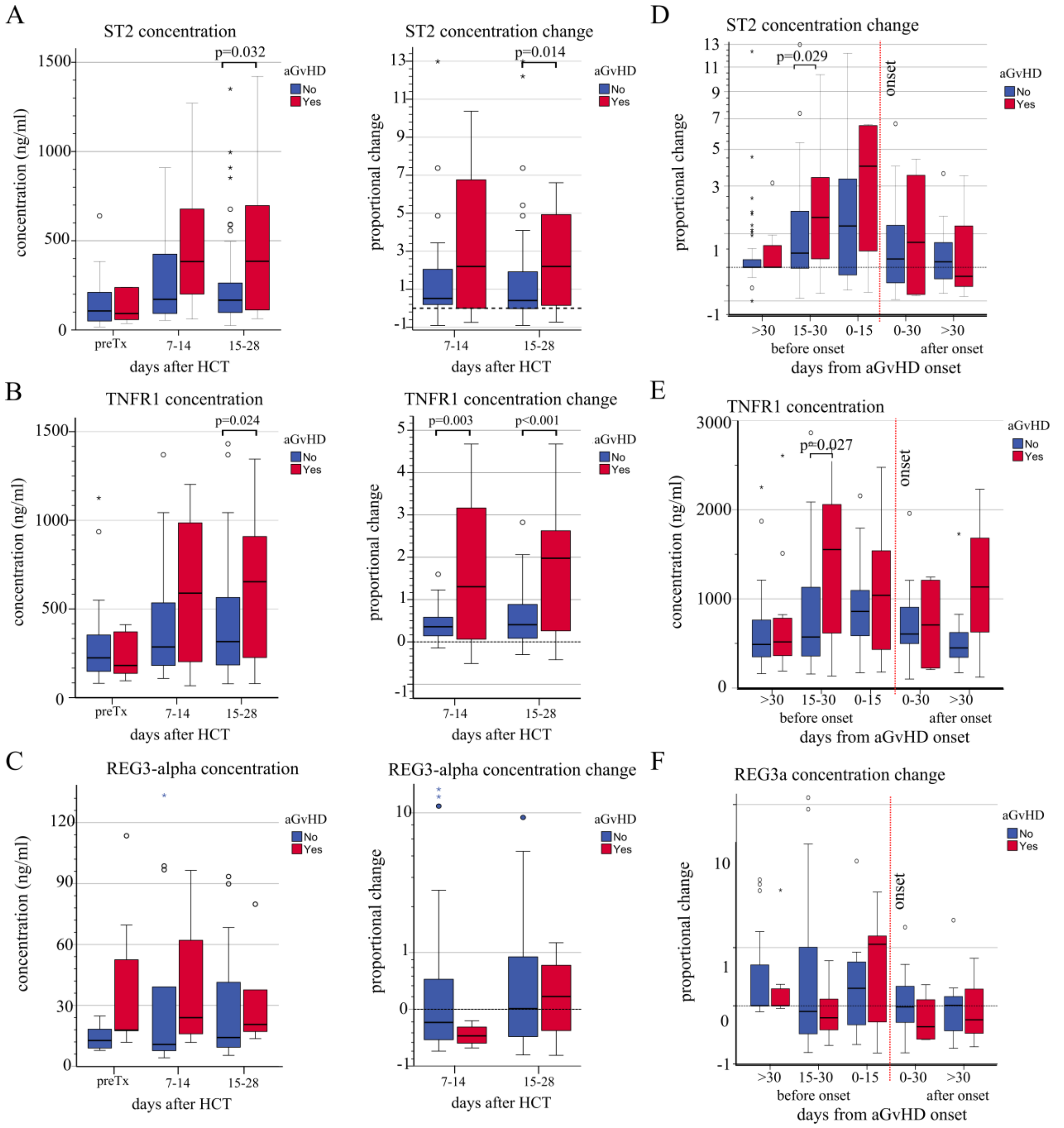

Supplementary Figure 5

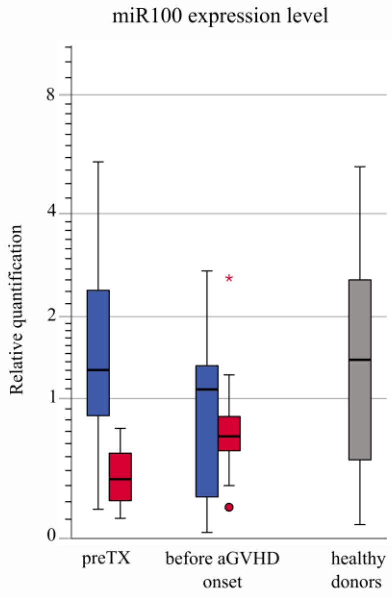

Supplement: Supplementary file 1 [file DataSheet_1.pdf]
